# Arginine Metabolism and Adenosine Receptor Signals in the Cerebellum Contribute to Nicotine Withdrawal‐Induced Anxiety/Depression‐Like Behaviours

**DOI:** 10.1111/adb.70076

**Published:** 2025-07-30

**Authors:** Wenjuan Zhang, Yu Tian, Xiao Yang, Baojiang He, Haifeng Zhang, Qi Zhang, Yingwu Mei

**Affiliations:** ^1^ Beijing Life Science Academy (BLSA) Beijing China; ^2^ Key Laboratory of Tobacco Flavor Basic Research of CNTC Zhengzhou Tobacco Research Institute of CNTC Zhengzhou Henan China; ^3^ Shanxi Kunming Tobacco Co. Ltd. Taiyuan China; ^4^ Department of Biochemistry and Molecular Biology, School of Basic Medical Sciences Zhengzhou University Zhengzhou Henan China

**Keywords:** adenosine receptor, arginine metabolism, cerebellum, nicotine withdrawal symptoms, purine metabolism, theobromine

## Abstract

Recent studies have established a strong association between the cerebellum and various psychiatric disorders, as well as drug addiction and withdrawal processes. However, the mechanisms underlying the cerebellum's role in nicotine withdrawal symptoms have yet to be explored. In this study, we employed transcriptome sequencing, untargeted metabolomics and integrative multi‐omics analysis to elucidate the molecular mechanisms underlying nicotine withdrawal‐induced affective symptoms, specifically anxiety and depression‐like behaviours, within the cerebellum. Our findings demonstrate that enhanced purine metabolism and disrupted arginine metabolism in the cerebellum significantly contribute to the development of anxiety and depression‐like behaviours in mice undergoing nicotine withdrawal. Treatment with the non‐selective adenosine receptor antagonist, theobromine, markedly alleviates these behaviours. This mechanism likely involves inhibiting adenosine signalling and restoring arginine metabolism in the cerebellum.

## Graphical abstract

This research indicates that enhanced purine metabolism and disrupted arginine metabolism contribute to the nicotine withdrawal‐induced anxiety and depression‐like behaviours in the cerebellum. Treatment with theobromine markedly alleviates these behaviours. The mechanism likely involves inhibiting adenosine signalling and restoring cAMP‐PKA, NO‐cGMP signalling and arginine metabolism in the cerebellum.

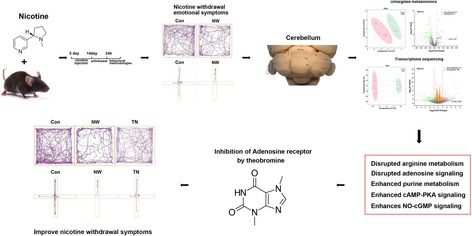

## 1 Introduction

Different from traditional understanding, recent studies have established a strong association between the cerebellum and various psychiatric disorders, including depression [1, 2] and anxiety disorders [3, 4]. Furthermore, emerging research also indicates the cerebellum's involvement in drug addiction and withdrawal processes. For instance, ethanol withdrawal is known to damage neurons and mitochondria in the cerebellum, leading to the excessive release of glutamate [5]. Additionally, the cerebellum plays a role in the development and withdrawal of opioid and cocaine addiction [6, 7]. Nicotine, the primary addictive component of tobacco, induces anxiety and depression‐like behaviours upon withdrawal, posing a significant barrier to cessation efforts [8, 9, 10]. Till now, the cerebellum's role in nicotine withdrawal remains unexplored.

Studies have shown that abnormal neurotransmitter release, such as dopamine, serotonin and norepinephrine, is crucial in anxiety and depression disorders [11, 12, 13]. These abnormalities in neurotransmitter synthesis are closely linked to changes in amino acid metabolism [14, 15]. For example, perturbations in glutamate, cysteine, methionine, arginine and proline metabolism in the prefrontal cortex have been linked with depressive behaviour [15]. Additionally, L‐arginine supplementation has demonstrated significant anti‐stress effects [16, 17]. Arginine metabolism is also intimately connected to NO signalling, with NO being synthesized by a family of nitric oxide synthases (NOS) by converting arginine to citrulline [18]. NO regulates the release of neurotransmitters, including norepinephrine, serotonin, dopamine and glutamate, and impacts the development of depression [19]. Neuronal NOS (nNOS, Nos1) is the predominant NOS isoform in the central nervous system (CNS). nNOS‐containing neurons are distributed throughout many CNS regions, with a notable presence in the hippocampus and cerebellum [20]. Emerging research has highlighted the cerebellum's role in emotional regulation, potentially linked to its NO signalling pathways [21, 22].

Adenosine signalling is known to regulate a variety of biological functions and play a role in numerous conditions [23]. In the cerebellum, adenosine modulates the release of glutamate neurotransmitters through its four receptors: A1, A2a, A2b and A3. This regulation impacts physiological functions such as movement and emotion [24]. Adenosine receptor activation predominantly enhances downstream cAMP‐PKA and NO‐cGMP signalling pathways [25]. Activation of A2a receptors has been confirmed to be associated with depressive behaviour [26, 27], although there is no consensus on the relationship between A1 receptor and depressive behaviour [23, 28, 29]. Additionally, adenosine signalling is also linked to drug addiction and withdrawal. Chronic drug exposure can alter adenosine metabolism in the nucleus accumbens [30]. Administration of an adenosine A1 receptor agonist can mitigate withdrawal behaviours and elevate dopamine levels during opioid withdrawal [31].

In this study, we utilized animal ethology, transcriptome sequencing and untargeted metabolomics to explore the correlation between anxiety and depression‐like behaviours induced by nicotine withdrawal and changes in cerebellar metabolites, aiming to analyse the underlying mechanisms. Our data indicate that enhanced purine metabolism and disrupted arginine metabolism significantly contribute to the development of anxiety and depression‐like behaviours in mice undergoing nicotine withdrawal. Treatment with the non‐selective adenosine receptor antagonist, theobromine, markedly alleviates these behaviours. The mechanism likely involves inhibiting adenosine signalling and restoring cAMP‐PKA, NO‐cGMP signalling and arginine metabolism in the cerebellum.

## 2 Materials and Methods

(S)‐(−)‐Nicotine (N412450) was obtained from Toronto Research Chemicals (Toronto, Canada), dissolved and diluted to the desired concentration through a physiological saline solution. Theobromine (T45040, 99% purity) was obtained from Shanghai Acmec Biochemical Technology Co. Ltd. All other reagents were of analytical grade and were commercially available.

### 2.1 Animals and Drug Treatment

Male C57BL/6J mice (aged 7–8 weeks) were obtained from the Charles River Laboratories (Beijing, China). The experimental animals were housed in cages under a 12 h light/12 h dark cycle, 60% ± 5% humidity, and a temperature of 25°C ± 1°C with access to water and food freely. All experimental procedures were conducted in accordance with the Ethics Committee of Zhengzhou University (ZZUIRB‐2024‐167).

### 2.2 Model of Nicotine Withdrawal

Building on previous research, we established a stable nicotine withdrawal model [9, 32, 33]. In brief, 20 adult mice were divided into two groups: a control group (Con) and a nicotine withdrawal group (NW), with 10 mice in each group. After a 7‐day acclimation period, the NW group received subcutaneous injections of nicotine at 2 mg/kg four times daily for 14 days, whereas the control group received an equal volume of saline. Following a 24‐h cessation of nicotine injections, behavioural tests were conducted on the mice. Then, the mice were euthanized via overdose anaesthesia, and their brains were perfused with cold PBS and dissected to obtain cerebellar tissue. The tissue samples were equally divided, with one portion subjected to transcriptome sequencing (RNA‐seq) and the other to untargeted metabolomics analysis, or used for other experiments.

### 2.3 Theobromine Treatment for Nicotine Withdrawal

Thirty adult mice were divided into three groups: a control group (Con), a nicotine withdrawal group (NW) and a theobromine treatment group (TN), with each group comprising 10 mice. The model establishment followed the same procedure as above. However, the TN group received a daily oral administration of 100 mg/kg theobromine (a mixture with 0.5% sodium carboxymethyl cellulose) in addition to nicotine injections. The dosage of the chemical compound was determined based on previous literature [34], and the Con and NW groups were given the same amount of 0.5% sodium carboxymethyl cellulose without theobromine.

### 2.4 Animal Behavioural Methodologies

All animal behaviour studies used equipment fitted with cameras and ANY‐maze 5.0 software. Open field test (OFT) and elevated plus maze (EPM) are used to evaluate anxiety‐like behaviour, whereas tail suspension test (TST) and forced swim test (FST) are used to evaluate depression‐like behaviour.

#### 2.4.1 OFT

To assess the locomotor activity and anxiety‐like behaviour of mice, a plain, 44 × 44 × 30 cm open field arena was utilized. After a 30‐s habituation period, the total distance travelled and the time spent in the centre and corner arenas (14.7 × 14.7 cm) were recorded during a 5‐min session.

#### 2.4.2 EPM

Anxiety‐like behaviour in mice was evaluated using the EPM test. The apparatus consists of two closed arms (30 × 6 × 15 cm) and two open arms (30 × 6 cm). Mice were allowed to explore the maze for 5 min, and the time spent in the closed and open arms, as well as the centre, was recorded.

#### 2.4.3 TST

Mice were suspended by their tails using adhesive tape, positioned 1 cm from the tip of the tail and 15 cm above the ground. Small plastic tubes were attached to their tails to prevent climbing. The test lasted for 6 min, and immobility time was measured and analysed during the final 4 min.

#### 2.4.4 FST

Individual mice were placed in a transparent glass cylinder (15 cm in diameter, 30 cm in height) filled with water (23°C ± 1°C) to a depth of 15 cm for 6 min. Immobility time was calculated during the last 4 min of the test.

### 2.5 RNA‐Seq and Bioinformatic Analysis

The quality of RNA was assessed using the Agilent 2100 Bioanalyzer System prior to library preparation. Libraries for sequencing were constructed following the protocol outlined in the NEBNext Ultra RNA Library Prep Kit for Illumina manual. These libraries were sequenced using 150‐bp paired‐end reads on an Illumina HiSeq platform (Novogene Co. Ltd., Beijing, China). Cleaned reads from each sample were aligned to the reference genomes (mouse: GRCm38) using HISAT2 software with default parameters. Gene read counts were then calculated using the Genomic Features and Genomic Alignments packages in R. Differential expression analysis and significance tests for genes were performed using the DESeq2 package. Pathway enrichment analysis using the Kyoto Encyclopedia of Genes and Genomes (KEGG) was conducted using the R package clusterProfiler.

### 2.6 Untargeted Metabolomics Analysis

Sample preparation followed established protocols as described in previous literature. Separation was achieved using liquid chromatography–mass spectrometry (LC‐MS) with a UHPLC System (1290, Agilent Technologies, Santa Clara, California, USA), equipped with an ACQUITY UPLC BEH C18 Column (Waters Corporation, Shanghai, China). The mobile phase consisted of 25 mmol/L ammonium acetate and 25 mmol/L ammonium hydroxide in a mixture of water and acetonitrile. An AB SCIEX Triple TOF 6600 was utilized for MS/MS spectra acquisition via information‐dependent acquisition during LC‐MS experiments. In this mode, the Analyst TF 1.7 (AB Sciex) continuously evaluated full‐scan mass spectrometry data and triggered the acquisition of MS/MS spectra based on predetermined criteria. During each cycle, the 12 precursor ions with intensities greater than 100 were selected for MS/MS analysis at a collision energy of 30 eV.

Mass spectrometry raw data files were converted to the mzXML format using ProteoWizard and processed using the R package XCMS (V.3.2). Processing included peak deconvolution, alignment and integration, with parameters set to Minfrac = 0.5 and cut‐off = 0.3. Identification of metabolites was performed using an in‐house MS2 database.

### 2.7 Immunohistochemistry

6 μm paraffin‐embedded cerebellums were de‐paraffinized, rehydrated and heat‐induced epitope retrieval and blocking of endogenous peroxidase activity. Then, sections were incubated with antibodies to Arg II, Asl and Assl (Proteintech, Wuhan, China). The DAB Peroxidase (HRP) Substrate Kit (BOSTER, Wuhan, China) was used to visualize positive staining. Sections were counterstained with haematoxylin to visualize nuclei. Images were captured using an Olympus microscope BX43.

### 2.8 Western Blotting

Samples were homogenized in RIPA buffer with a protease inhibitors cocktail on ice for 30 min. The lysates were centrifuged at 13 000 g for 10 min at 4°C. The supernatant was collected, aliquoted and stored at −80°C until use. The protein concentration of each sample was determined by BCA assay. Samples were separated on 10% SDS‐PAGE and then transferred by electroblotting to a nitrocellulose membrane. The membrane was blocked in 5% BSA and then incubated with primary antibodies against nNOS, iNOS, eNOS, A1 receptor, A2a receptor, A2b receptor, A3 receptor, Arg II, PKA, p‐PKA and GAPDH (Santa Cruz, Shanghai, China) in 2% BSA overnight at 4°C. After washing, the membrane was incubated with horseradish peroxidase–labelled secondary antibodies for 1 h at room temperature. Bands were visualized using a BeyoECL Plus kit (Beyotime, Shanghai, China).

### 2.9 Real‐Time Quantitative PCR

Cerebellar samples from different groups were used for gene expression analysis by a quantitative real‐time polymerase chain reaction. Amplification and detection using TIANGEN Talent qPCR PreMix (FP209, Beijing, China)were performed with an ABI Prism 7500 sequence detection system (Applied Biosystems, Foster City, CA, USA). All the gene's primers are given in Table S1.

### 2.10 NO, cAMP and cGMP Assay

The levels of nitric oxide, cAMP, and cGMP in plasma and cerebellar tissue were detected using commercial reagent kits, the Nitric Oxide Assay Kit (Thermo Fisher, Shanghai, China), cAMP Assay Kit, and cGMP Assay Kit (Abcam, Shanghai, China). All samples were homogenized under the protection of argon gas in the modified RIPA buffer (50 mM Tris‐HCl, pH 8.0, 150 mM NaCl, 1% NP‐40 and 0.5% Triton X‐100), then centrifuged at 13 000 rpm, 4°C for 10 min. The supernatant was stored and tested according to the instructions of the reagent kit.

### 2.11 Statistical Analysis

The data were presented as mean ± standard error of the mean (SEM). Statistical significance was determined using a two‐tailed unpaired Student's *t*‐test for comparisons between two groups or one‐way ANOVA for comparisons among multiple groups, and the normal distribution test was performed before these tests. For datasets analysed with ANOVA, the Bonferroni post hoc test was used. A *p* value less than 0.05 was considered statistically significant. Statistical analyses were performed using Prism Version 8.0 (GraphPad, La Jolla, CA, USA).

## 3 Results

### 3.1 Nicotine Withdrawal Causes Significant Anxiety/Depression‐Like Behaviours and Metabolites Changes in the Cerebellum

**FIGURE 1:**
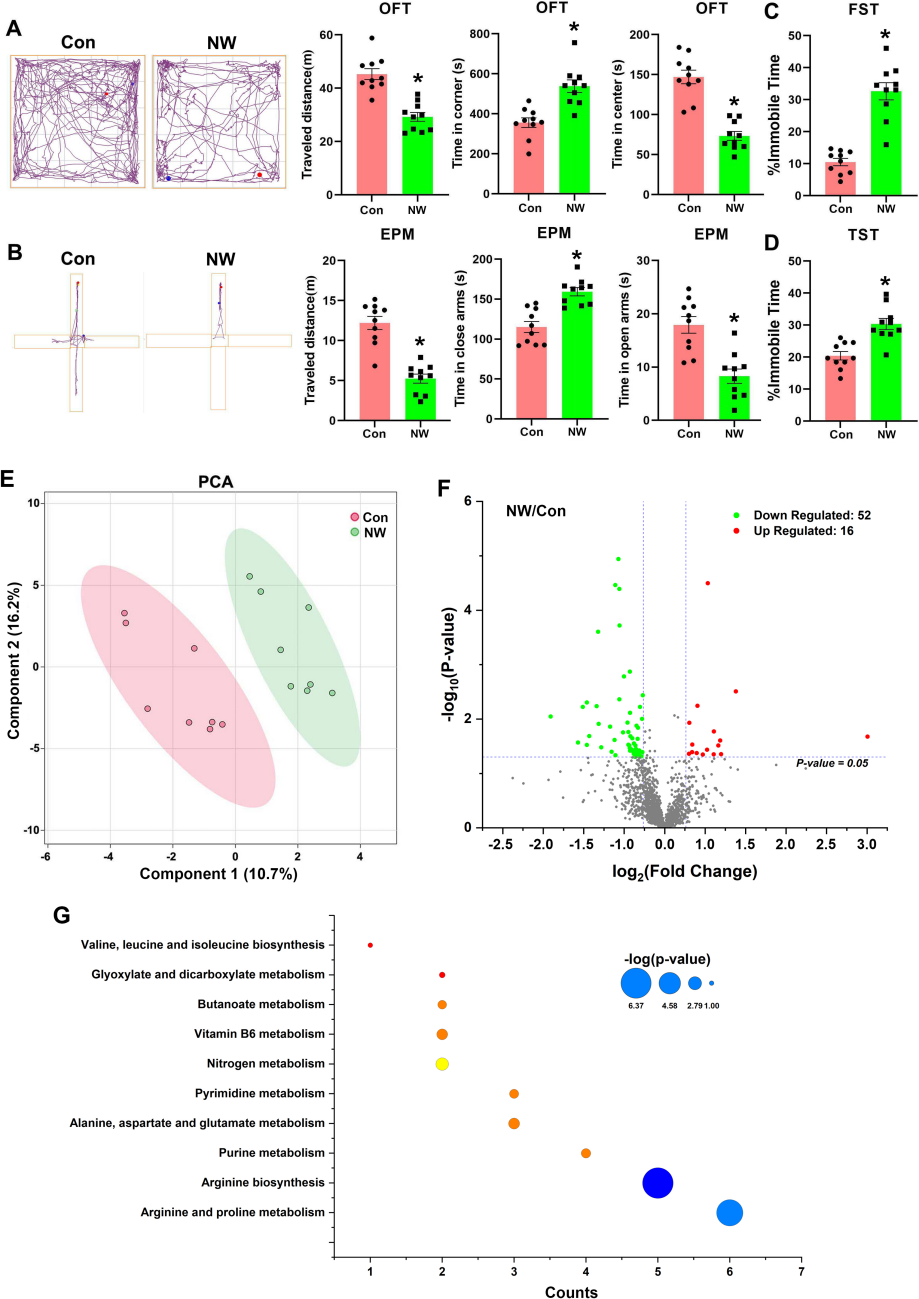
Nicotine withdrawal induces notable anxiety and depression‐like behaviours and triggers substantial metabolite changes in the cerebellum. (A) An open field test (OFT) was conducted to assess anxiety‐like behaviours following nicotine withdrawal, measuring the time spent in corners and the centre, as well as the animal's movement distance. (B) An elevated plus maze (EPM) was utilized to evaluate anxiety‐like behaviours after nicotine withdrawal, recording the animal's movement distance and the time spent in the closed arms and open arms, respectively. (C,D) The forced swimming test (FST) and tail suspension test (TST) were employed to detect depressive‐like behaviours following nicotine withdrawal, with the percentage of immobility time serving as an indicator of the severity of depressive symptoms. (E) Principal component analysis (PCA) of metabolites changes in cerebellum between the control (Con) and NW groups from untargeted metabolomics data. (F) Volcano plot showing differential metabolite changes between the Con and NW groups, and the differential multiples were more than 1.5. The metabolites with significant changes are coloured. The dotted lines indicate the cut‐off for significant changes (*p* < 0.05). (G) KEGG functional enrichment analysis for the differential metabolites. All the data are expressed as mean ± SEM (*n* = 10 in behaviours test and *n* = 8 in untargeted metabolomics analysis per group), *p < 0.05, Con versus NW.

In our study, we assessed anxiety and depression‐like behaviours 24 h after terminating nicotine injections according to previous literature [9]. As anticipated, the NW mice spent less time in the centre and more time in the corners during the OFT (Figure 1A, *p* = 0.0002 and *p* < 0.0001), and they also spent less time in the open arms and more time in the closed arms of the EPM compared to the control mice (Figure 1B, *p* < 0.0001 and *p* = 0.0002). Additionally, in both the OFT and EPM experiments, the NW mice demonstrated reduced locomotor activity, covering less distance than the control mice (Figure 1A,B, *p* < 0.0001 and *p* < 0.0001). These observations indicate that nicotine withdrawal induces significant anxiety‐like behaviours in mice. Furthermore, in the TST and the FST, the duration of immobility, which reflects the level of behavioural despair, was significantly increased in NW mice compared to controls. This increase in immobile time suggests that NW mice exhibit more pronounced depression‐like behaviours (Figure 1C,D, *p* = 0.0002 and *p* < 0.0001). In summary, our animal model demonstrates significant affective symptoms associated with nicotine withdrawal, providing a robust platform for further investigation into the underlying mechanisms.

In the untargeted metabolomics, we found nicotine withdrawal induced significant metabolic changes in the cerebellum. Principal component analysis (PCA) of the untargeted metabolomics data revealed distinct clustering, signifying substantial metabolic differences between NW mice and control mice (Figure 1E). The volcano plot underscored small molecules with a fold change greater than 1.5, identifying 52 metabolites with a significant increase and 16 metabolites with a significant decrease in their levels (Figure 1F). KEGG enrichment analysis of these differentially abundant metabolites revealed predominant enrichment in pathways related to amino acid metabolism and nitrogen‐containing compound metabolism (Figure 1G). Specifically, the top three enriched pathways were arginine and proline metabolism, arginine biosynthesis and purine metabolism.

### 3.2 The Anxiety/Depression‐Like Behaviours Caused by Nicotine Withdrawal Are Closely Related to Arginine Metabolism and Purine Metabolism

**FIGURE 2:**
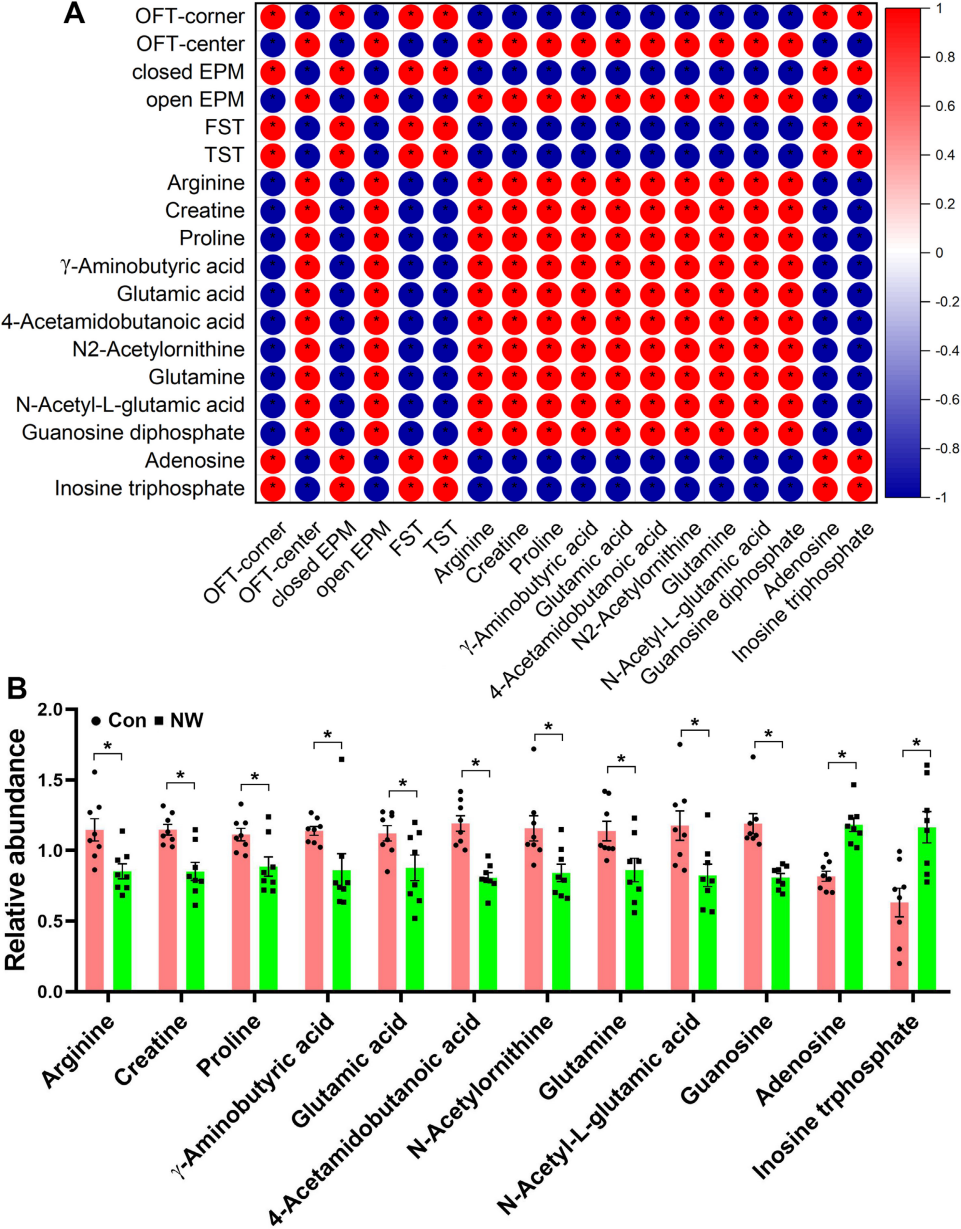
The anxiety/depression‐like behaviours caused by nicotine withdrawal are closely related to arginine metabolism and purine metabolism. (A) Correlation analysis between anxiety/depression‐like behaviours and differential metabolites identified in the arginine and proline metabolism, arginine biosynthesis and purine metabolism. (B) The relative content of identified 12 differential metabolites in the cerebellum. All the data are expressed as mean ± SEM (*n* = 8 per group in the changed metabolites), *p < 0.05, Con versus NW.

Untargeted metabolomics analysis has revealed that changes in cerebellar metabolites due to nicotine withdrawal are predominantly enriched in the arginine and proline metabolism, arginine biosynthesis and purine metabolism pathways. Moving forward, we will precisely analyse the correlation between anxiety/depression‐like behaviours and the differential metabolites associated with these three metabolic pathways (Figure 2A). A total of 12 differential metabolites were identified within these pathways. Specifically, arginine, creatine, proline, γ‐aminobutyric acid, glutamic acid and 4‐acetamidobutanoic acid are part of the arginine and proline metabolism pathway; the levels of these metabolites are negatively correlated with anxiety and depressive‐like behaviours. In the arginine biosynthesis pathway, arginine, glutamic acid, N2‐acetylornithine, glutamine and N‐acetyl‐L‐glutamic acid levels are also negatively correlated with anxiety and depressive‐like behaviours. Within the purine metabolism pathway, adenosine and inosine triphosphate (ITP) are positively correlated with anxiety and depressive‐like behaviours, whereas guanosine diphosphate is negatively correlated with these behaviours.

Comparing the levels of these 12 metabolites, we found that, except for the increased levels of adenosine and ITP, all other metabolites were significantly decreased in the NW group (Figure 2B; *F*_1,168_ = 37.40, *p* < 0.0001). The literature indicates that arginine metabolism is associated with depression, and supplementing arginine can enhance stress resistance [16, 17]. Additionally, the adenosine signalling pathway has been shown to regulate anxiety and depression behaviours, with activation of A2a receptors exacerbating depressive‐like symptoms [35]. Therefore, based on our metabolomics results, we infer that the affective symptoms of nicotine withdrawal may be linked to alterations in arginine metabolism and adenosine signalling in the cerebellum.

### 3.3 RNA‐Seq Analysis Showed Nicotine Withdrawal‐Induced Changes in Arginine Metabolism, cAMP and cGMP Signalling Pathways in the Cerebellum

**FIGURE 3:**
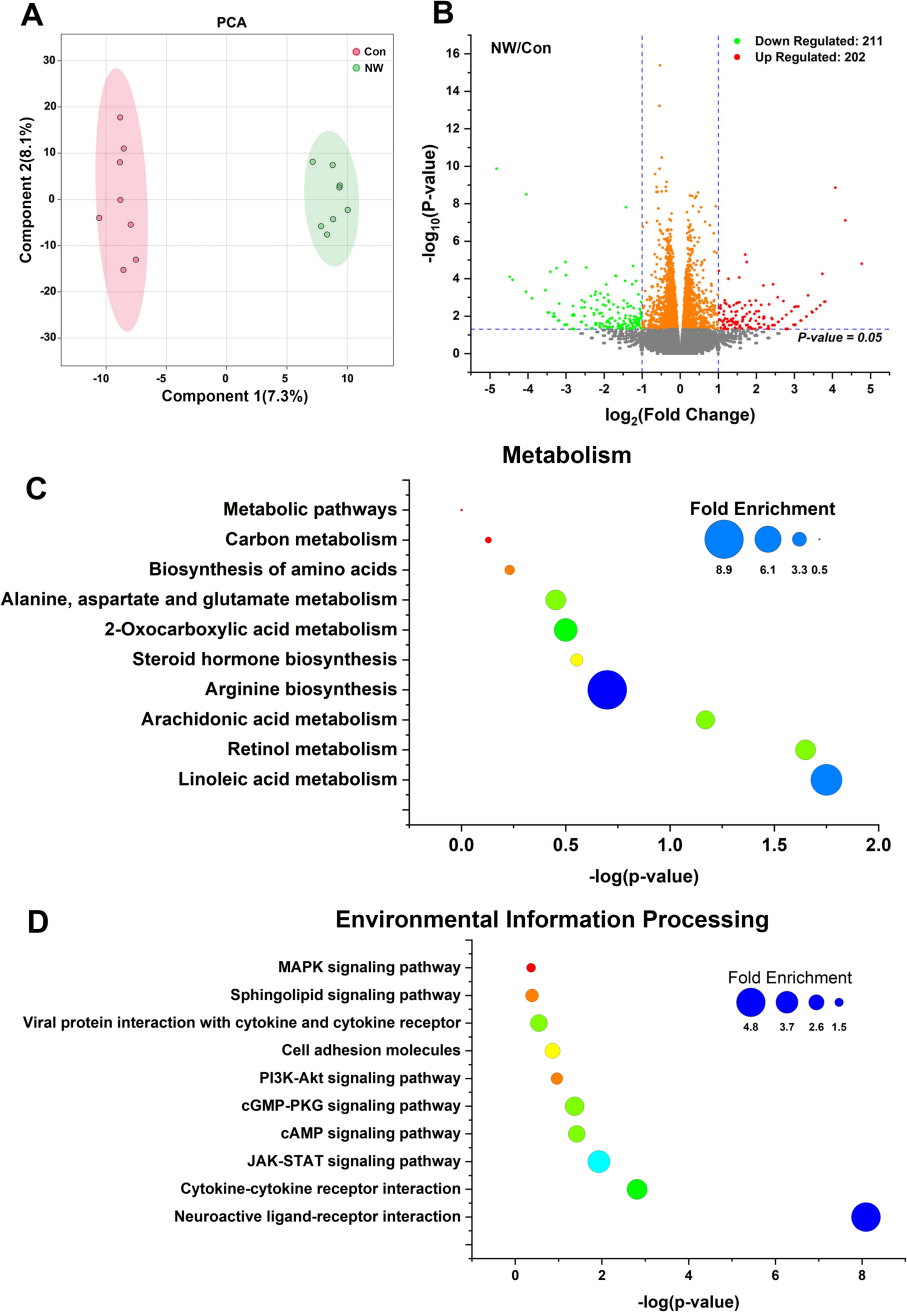
RNA‐seq analysis of gene expression changes in the cerebellum after nicotine withdrawal. (A) Comparison of RNA‐seq gene expression in the cerebellum between the Con group and NW group through principal component analysis (PCA). (B) Volcano plot showing differential gene expression between the Con group and NW group, and the differential expression multiples were more than 2. The genes with significant changes are coloured. The dotted lines indicate the cut‐off for significant changes (*p* < 0.05). (C) Signal pathways belonging to metabolism subclass in KEGG enrichment analysis of significantly differential genes. (D) Signal pathways belonging to environmental information processing subclass in KEGG enrichment analysis of significantly differential genes (*n* = 8 per group in the RNA‐Seq).

In the next, we conducted transcriptome sequencing to explore changes in gene expression levels induced by nicotine withdrawal. As shown in Figure 3, the PCA effectively grouped the data into two distinct clusters, indicating substantial transcriptional changes in the NW mice. The volcano plot indicates that nicotine withdrawal caused changes in 413 genes in the cerebellum (*p* < 0.05, fold change > 2), with 211 genes upregulated and 202 genes downregulated. KEGG enrichment analysis of these differentially expressed genes revealed that they were mainly distributed across five categories: human diseases, organismal systems, environmental information processing, metabolism and cellular processes (Figure S1). Within metabolism, the main enriched pathways included linolenic acid metabolism, retinol metabolism, arachidonic acid metabolism and arginine metabolism. In environmental information processing, the key enriched pathways included neuroactive ligand–receptor interaction, cytokine–cytokine receptor interaction, cAMP signalling and cGMP‐PKG signalling.

Based on the metabolomics data, we focused on arginine metabolism and the cAMP and cGMP signalling pathways and conducted a joint analysis (Figure S2). The results showed that all metabolites and genes involved in the arginine biosynthesis and arginine–proline metabolism pathways were significantly reduced in the NW group (indicated by a green background). In the cAMP and cGMP signalling pathways, the most notable finding was the involvement of the adenosine A1 and A2a receptors in signal transduction. All metabolites and genes associated with the A2a receptor transduction pathway were upregulated (red background), whereas those associated with the A1 receptor pathway were downregulated (green background). γ‐Aminobutyric acid (C00334) and oxytocin (Oxt), which are upstream molecules of the adenosine A1 receptor, have been shown to have significant inhibitory effects on anxiety and depression [36, 37]. Activation of the A2a receptor is known to promote anxiety and depression, and its antagonists have demonstrated significant anti‐anxiety and anti‐depressive effects [38].

Therefore, comprehensive data from untargeted metabolomics and transcriptome sequencing suggest that the affective symptoms of nicotine withdrawal may be associated with disrupted arginine metabolism and enhanced adenosine signalling in the cerebellum.

### 3.4 nNOS and NO Link Arginine Metabolism With Adenosine Signalling Pathways and Play Important Roles in Anxiety/Depression‐Like Behaviours Caused by Nicotine Withdrawal

**FIGURE 4:**
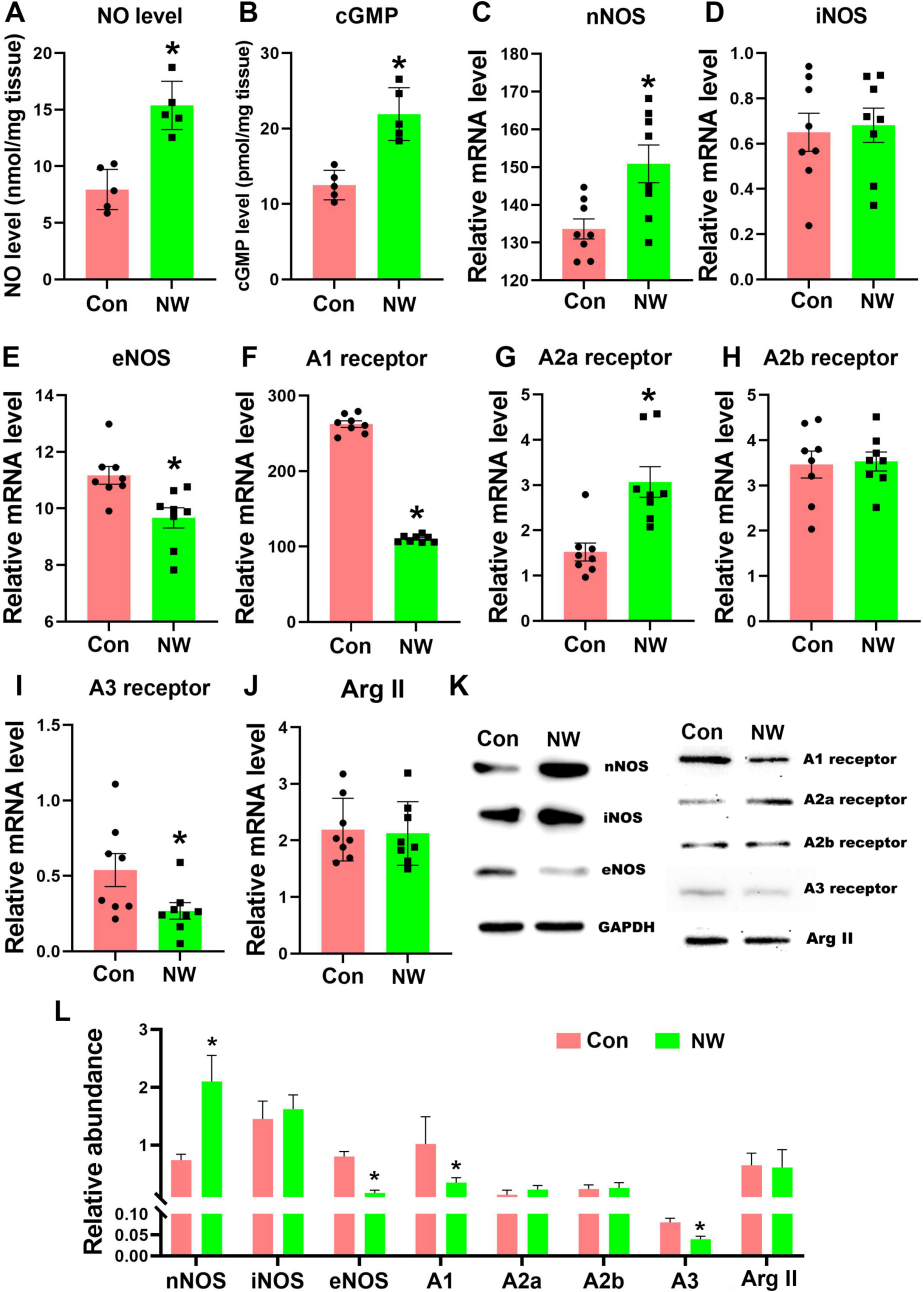
Adenosine receptors and the NO‐cGMP signalling pathway are involved in anxiety and depression‐like behaviours caused by nicotine withdrawal. (A) Comparison of NO levels in the cerebellum of mice from the control and NW groups. (B) Comparison of cGMP levels in the cerebellum of mice from the control and NW groups. (C–E) Relative mRNA levels of three types of nitric oxide synthase (nNOS, iNOS and eNOS) were detected by RT‐PCR. (F–I) Relative mRNA levels of four types of adenosine receptors (A1, A2a, A2b and A3) were detected by RT‐PCR. (J) Relative mRNA levels of arginase 2 detected by RT‐PCR. (K) Detection of protein expression of three nitric oxide synthases, four adenosine receptors and arginase 2 by western blot. (L) Relative grayscale values of western blot in (K). All the data are expressed as mean ± SEM (*n* = 5 in the NO and cGMP detection, *n* = 8 in RT‐PCR and *n* = 3 in western blot), *p < 0.05, Con versus NW.

Our multi‐omics analysis has revealed that nicotine withdrawal induces alterations in arginine metabolism, as well as the cAMP and cGMP signalling pathways in the cerebellum. NO and adenosine are the main upstream signalling molecules of the cAMP and cGMP signalling. We first measured the levels of NO and cGMP in the cerebellum, finding that both were significantly higher in the NW group compared to controls (Figure 4A,B; *p* < 0.0001 and *p* = 0.0008). This indicates that the cGMP signalling pathway is activated in the cerebellum of nicotine withdrawal mice. Elevated adenosine levels can activate endothelial NOS (eNOS) via its A1 receptor, further increasing NO levels. However, because neuronal NOS (nNOS) is the predominant NOS subtype in the cerebellum, the increased NO levels likely result from arginine breakdown by nNOS. To verify this, we assessed the expression levels of the three NOS types using RT‐PCR and western blotting. RT‐PCR results showed that nNOS expression was the highest in the cerebellum and significantly higher in the NW group compared to controls. In contrast, eNOS expression was significantly reduced in the NW group, whereas inducible NOS (iNOS) expression remained unchanged between the groups (Figure 4C–E; *p* = 0.0086, *p* = 0.79 and *p* = 0.0068). These results were corroborated by western blotting (Figure 4K,L; p_nNOS_ = 0.0192, p_eNOS_ = 0.0247 and p_A1_ = 0.0093). Arginine levels are primarily determined by its synthesis and degradation, with arginine synthase 2 (Arg II) being the main enzyme for arginine production in the cerebellum. The expression level of Arg II did not show significant changes (Figure 4J; *p* = 0.8081). Additionally, assessing adenosine receptor expression levels revealed that A1 and A3 receptors were significantly downregulated (Figure 4F,I; *p* < 0.0001 and *p* = 0.0432), whereas the A2a receptor was significantly upregulated (Figure 4G; *p* = 0.0014). The A2b receptor expression level remained unchanged (Figure 4H; *p* = 0.8592). These findings were also confirmed by western blotting (Figure 4K,L).

So our results indicate that nicotine withdrawal enhances adenosine A2a receptor signalling, inhibits A1 receptor signalling and activates the NO‐cGMP signalling pathway by increasing NO synthesis. The elevated NO levels are due to increased nNOS expression, which may also contribute to the observed decrease in cerebellar arginine levels.

### 3.5 Adenosine Receptor Inhibitor, Theobromine, Can Significantly Improve Nicotine Withdrawal‐Induced Anxiety/Depression‐Like Behaviours

**FIGURE 5:**
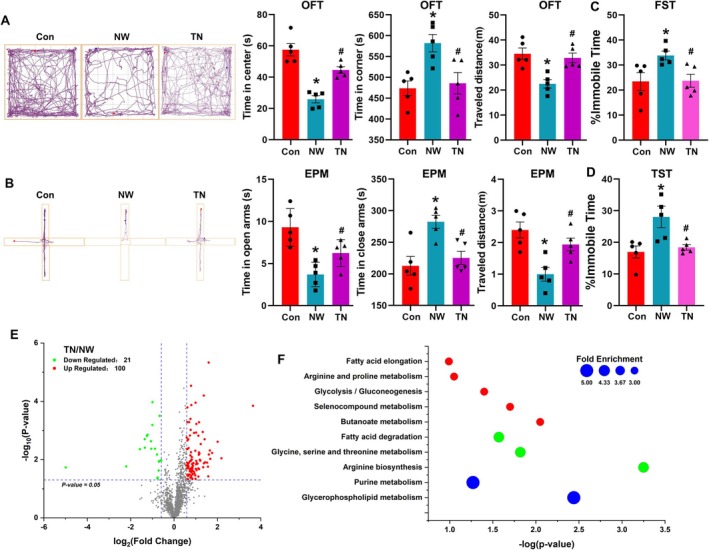
Adenosine receptor inhibition improves nicotine withdrawal‐induced anxiety/depression‐like behaviour and causes metabolite changes in the cerebellum. (A) An open field test (OFT) was conducted to compare the anxiety‐like behaviour in Con (control), NW (nicotine withdrawal) and TN (theobromine treatment) groups, measuring the time spent in corners and the centre, as well as the animal's movement distance. (B) An elevated plus maze (EPM) was utilized to evaluate anxiety‐like behaviour in Con, NW and TN groups, recording the animal's movement distance and the time spent in the closed arms and open arms, respectively. (C,D) The forced swimming test (FST) and tail suspension test (TST) were employed to detect depressive‐like behaviour in Con, NW and TN groups, with the percentage of immobility time serving as an indicator of the severity of depressive symptoms. (E) Volcano plot showing differential metabolite changes between NW and TN groups, and the differential multiples were more than 1.5. The metabolites with significant changes are coloured. The dotted lines indicate the cut‐off for significant changes (*p* < 0.05). (F) KEGG functional enrichment analysis for the differential metabolites. All the data are expressed as mean ± SEM (*n* = 5 in the behaviours test and *n* = 8 in untargeted metabolomics analysis per group), *p < 0.05, Con versus NW, #*p* < 0.05, NW versus TN.

To further determine the role of adenosine signalling in nicotine withdrawal, theobromine, an adenosine receptor inhibitor, was used to study the effects of adenosine receptor inhibition in this study. As shown in Figure 5A, NW mice spent less time in the centre and more time in the corners of the OFT compared to the control mice. However, when fed with theobromine (TN group), the mice spent more time in the centre and less time in the corners compared to NW mice (*F*_2,12_ = 9.557, *p* = 0.0033; *F*_2,12_ = 12.15, *p* = 0.0013). This pattern was also observed in the EPM (Figure 5B), where NW mice spent more time in the closed arms and less time in the open arms. In contrast, theobromine‐fed mice spent more time in the open arms and less time in the closed arms compared to the NW mice (*F*_2,12_ = 30.00, *p* < 0.0001; *F*_2,12_ = 7.518, *p* = 0.0076). Additionally, the total movement distance indicated that theobromine could restore the reduced motility caused by nicotine withdrawal (Figure 5A,B; *F*_2,12_ = 10.14, *p* = 0.0026; *F*_2,12_ = 10.77, *p* = 0.0021). The TST and FST showed that theobromine feeding eliminated depressive‐like behaviours caused by nicotine withdrawal (Figure 5C,D; *F*_2,12_ = 4.689, *p* = 0.0313, *F*_2,12_ = 6.952, *p* = 0.0099). Overall, data from these animal behavioural tests indicate that theobromine feeding ameliorated anxiety and depression‐like behaviours induced by nicotine withdrawal.

### 3.6 Inhibition of Adenosine Receptors Significantly Causes Changes in Cerebellar Metabolites, Particularly in Arginine and Purine Metabolism

**FIGURE 6:**
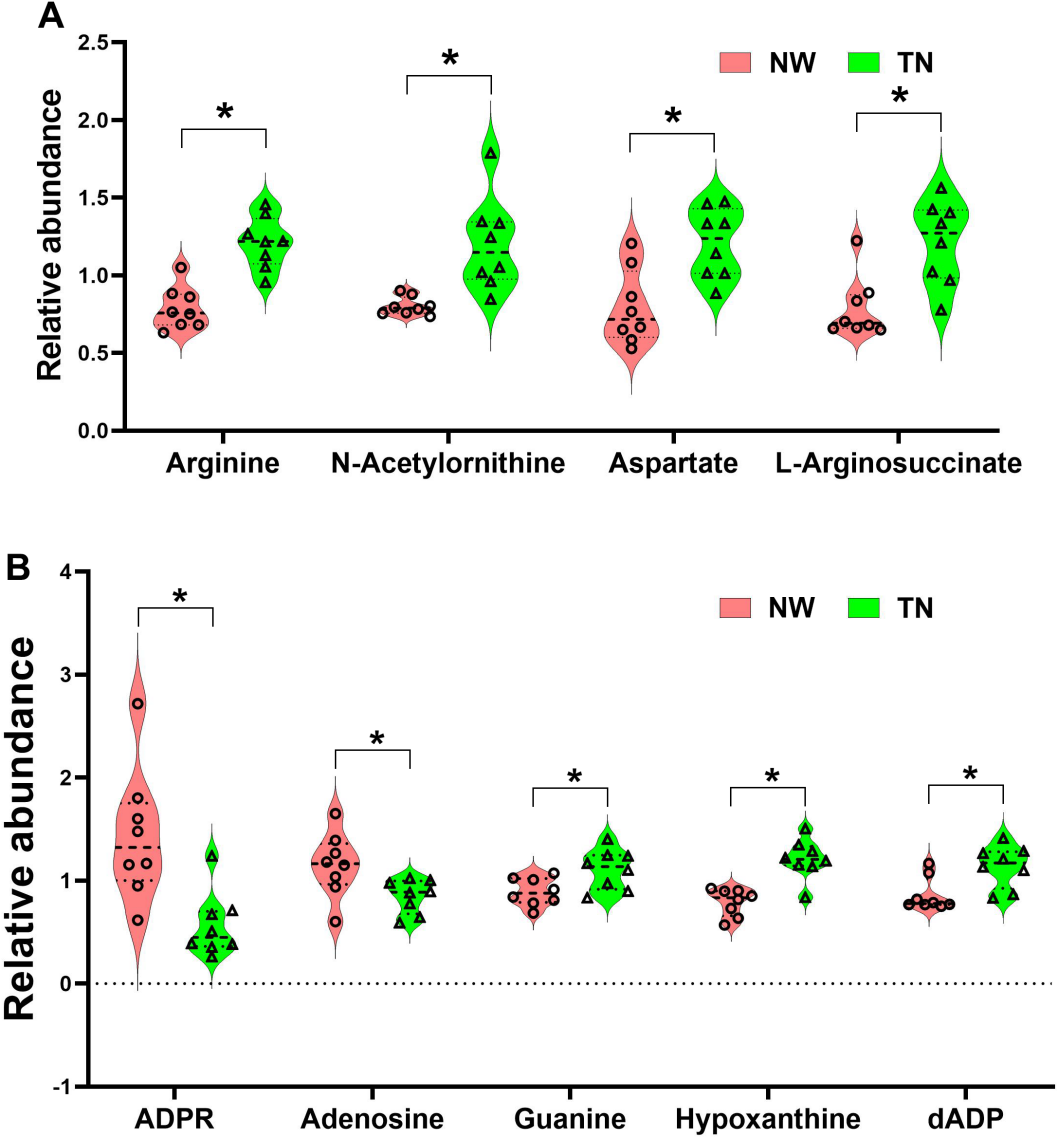
Differential metabolites detected by metabolomics belong to the arginine and purine metabolism pathway in the cerebellum after adenosine receptor inhibition. (A) Differential metabolites belong to the arginine metabolism pathway. (B) Differential metabolites belong to the purine metabolism pathway. All the data are expressed as mean ± SEM (*n* = 8 per group), *p < 0.05, NW versus TN.

To comprehensively explore the mechanism of inhibiting adenosine receptors causing physiological changes, we have once again applied untargeted metabolomics to study changes in small molecule metabolites in the cerebellum. Surprisingly, most of the metabolites with significant changes caused by theobromine showed an increase in content (Figure 5E). The volcano plot shows that out of 121 significantly changing metabolites (*p* < 0.05, fold change > 1.5), 21 metabolites were downregulated and 100 metabolites were upregulated. The main KEGG enrichment pathways of these metabolites are glycerophospholipid metabolism, purine metabolism, arginine synthesis, etc. There are four metabolites enriched in the arginine metabolism pathway: arginine, N‐acetylornithine, arginosuccinate and aspartate. The content of these metabolites is significantly higher in the TN group than in the NW group (Figure 6A; *F*_1,56_ = 61.59, *p* < 0.0001). This result shows that the adenosine receptor inhibitor, theobromine, significantly alters arginine metabolism. There are five metabolites with significant differences belonging to the purine metabolism pathway, among which ADPR and adenosine are significantly reduced in the TN group, whereas guanine, hypoxanthine and dADP are significantly increased in the TN group (Figure 6B; *F*_1,70_ = 0.6799, *p* = 0.4124). Because theobromine belongs to adenosine analogues, it may inhibit the de novo synthesis of adenosine through feedback inhibition.

**FIGURE 7:**
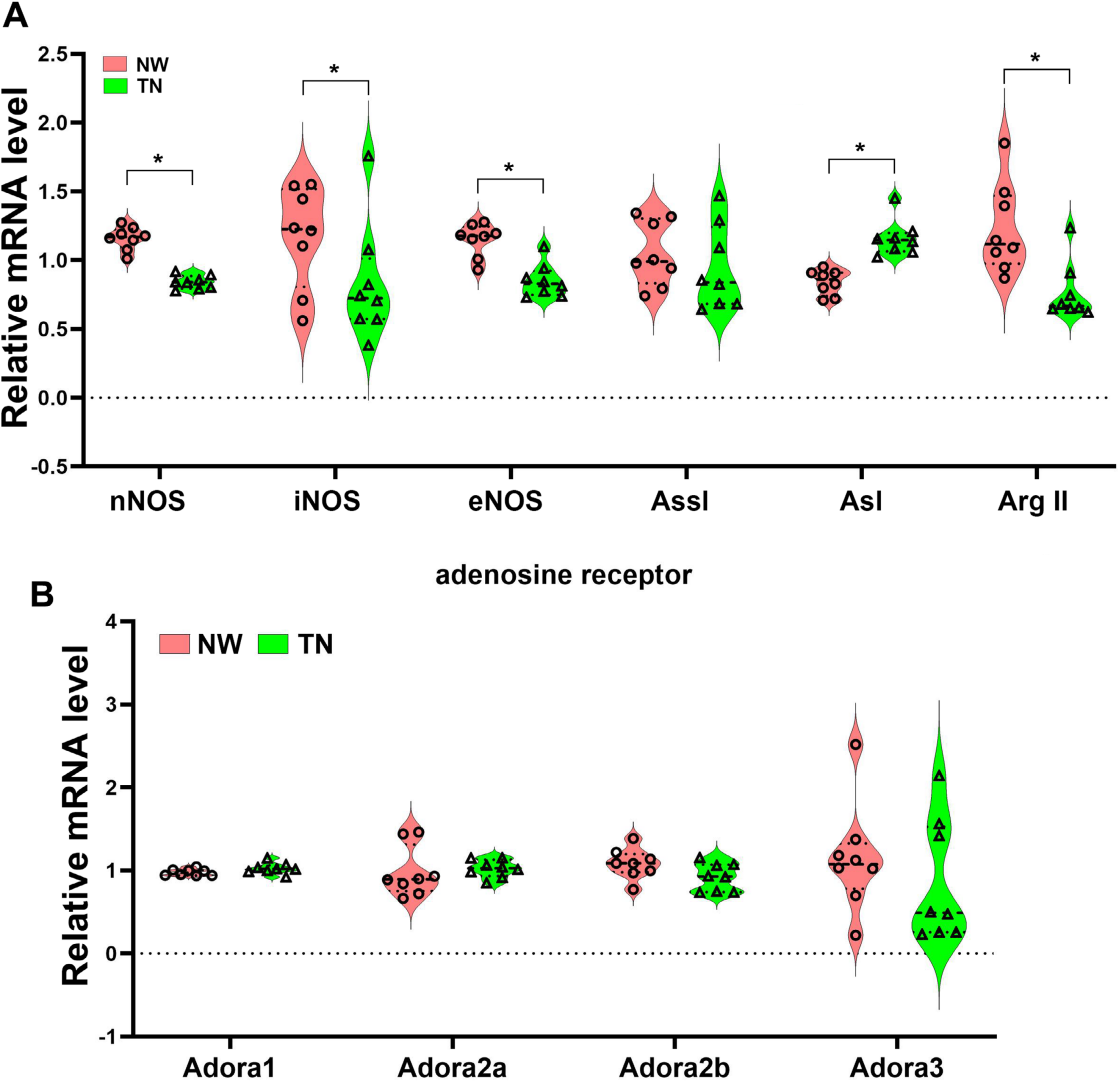
RT‐PCR detection of arginine metabolism‐related genes and adenosine receptor gene expression after adenosine receptor inhibition. (A) Comparison of gene expression levels related to arginine metabolism in the cerebellum. (B) Comparison of expression levels of four subtypes of adenosine receptors. All the data are expressed as mean ± SEM (*n* = 8 per group), *p < 0.05, NW versus TN.

We next used RT‐PCR to detect important molecules in arginine metabolism, such as three types of nitric oxide synthase (nNOS, iNOS, eNOS), arginase II (Arg II), argininosuccinate lyase (Asl) and argininosuccinate synthase (Assl) (Figure 7A; *F*_1,84_ = 17.03, *p* < 0.0001; Figure S4). The data showed that the mRNA levels of all nitric oxide synthase were reduced in the TN group, indicating that the nitric oxide signalling pathway may be weakened. Ass1 can catalyse the formation of L‐arginosuccinate from citrulline and aspartate. There was no difference in mRNA levels between nicotine withdrawal and theobromine treatment groups. Therefore, the increased levels of aspartate may be the reason for the increase in L‐arginosuccinate. Asl can cleave L‐arginosuccinate to produce fumarate and arginine, and its mRNA levels significantly increase in the TN group, which may be an important reason for the increase in arginine levels. Arg II is the main arginase expressed in the brain, and its mRNA level was significantly reduced in the TN group, indicating a weakening of arginine cleavage, which may be another reason for the increase in arginine content. In addition, we also tested the mRNA levels of adenosine receptors. It was found that the treatment with theobromine did not alter the transcription levels of the four adenosine receptors (Figure 7B; *F*_1,56_ = 0.9054, *p* = 0.3454).

### 3.7 Theobromine Inhibits Adenosine Receptor, Arginine Metabolism and NO‐cGMP Signalling Pathway to Improve Nicotine Withdrawal Emotional Symptoms

**FIGURE 8:**
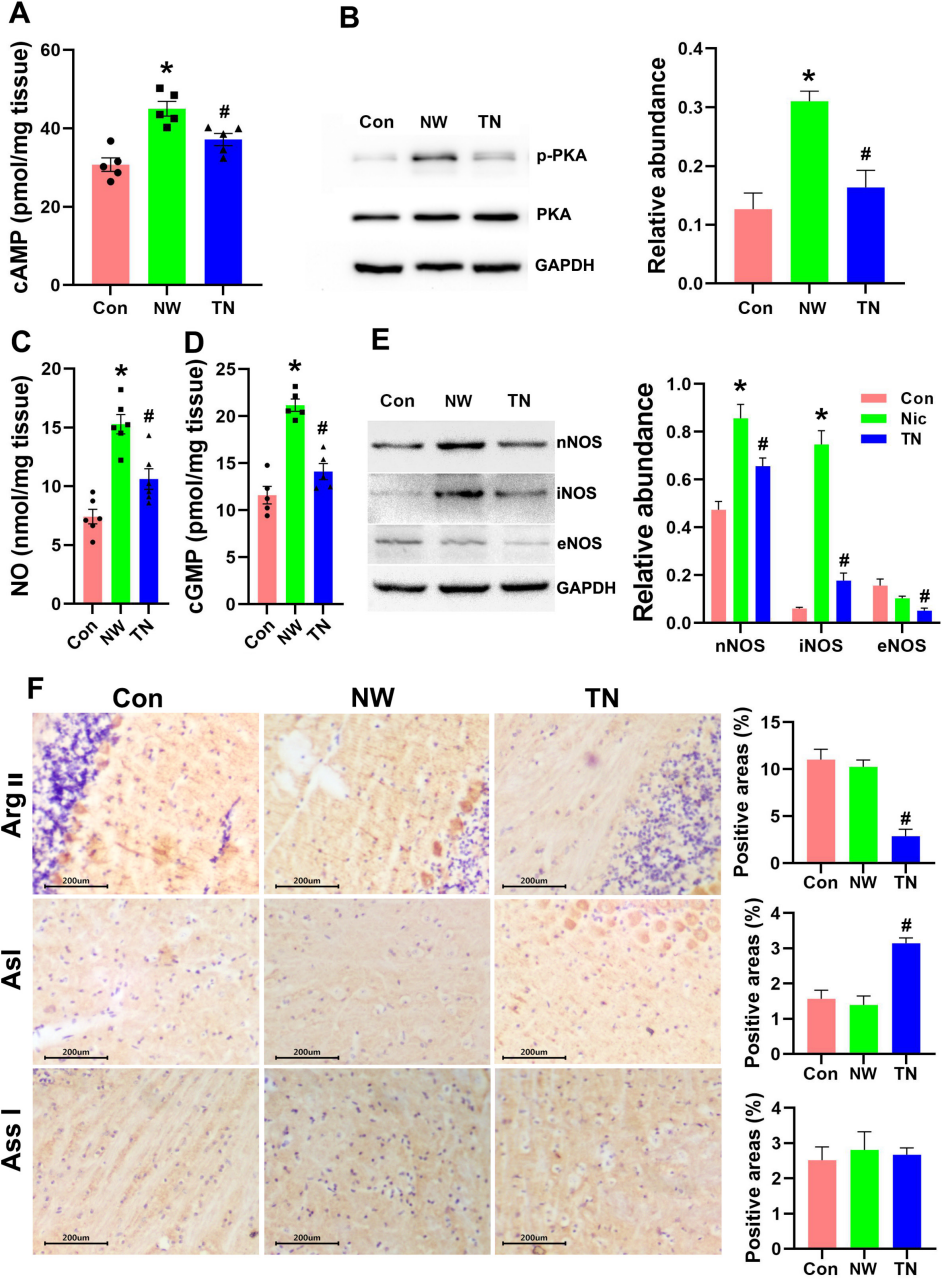
Adenosine receptor inhibition regulates arginine metabolism and restrains the NO‐cGMP signalling pathway to improve nicotine withdrawal emotional symptoms. (A) Comparison of cAMP levels in the cerebellum of mice from the control, NW and TN groups. (B) Detection of protein expression and phosphorylation levels of PKA by western blot. (C,D) Comparison of NO and cGMP levels in the cerebellum of mice in the control, NW and TN groups. (E) Detection of protein expression of three types of nitric oxide synthase by western blot. (F) Immunohistochemical detection of the expression level of arginase II (ArgII), argininosuccinate lyase (Asl) and argininosuccinate synthase (Ass1) from the control, NW and TN groups (*n* = 5 in cAMP, NO and cGMP detection and *n* = 3 in western blot and immunohistochemistry per group), *p < 0.05, Con versus NW, #*p* < 0.05, NW versus TN.

The cAMP‐PKA signalling pathway is pivotal in activating adenosine receptors [39]. Our findings revealed that cAMP and PKA phosphorylation levels were significantly elevated in the NW group, whereas these levels were markedly reduced when adenosine receptors were inhibited by theobromine (Figure 8A,B; *F*_2,12_ = 17.13, *p* = 0.0003, *F*_2,6_ = 14.95, *p* = 0.0047). These results suggest that the activation of adenosine receptor signalling in the cerebellum plays a critical role in the anxiety and depression‐like behaviours associated with nicotine withdrawal. Moreover, nicotine withdrawal was found to increase nitric oxide and cGMP levels in the cerebellum (Figures 4A,B and 8C,D; *F*_2,15_ = 25.65, *p* < 0.0001, *F*_2,12_ = 36.37, *p* < 0.0001); theobromine treatment partially restored these levels, indicating that theobromine suppresses both adenosine signalling and the NO‐cGMP signalling pathway in the cerebellum (Figure 8C,D). Additionally, western blot analysis showed that theobromine also inhibited the expression of all three types of NOS compared to the NW group (Figure 8E; *F*_2,18_ = 78.67, *p* < 0.0001). Arginine metabolism is a key factor in nitric oxide production. Important enzymes in arginine metabolism, including ArgII, Asl, Ass1 and the three NOS types, dictate tissue arginine levels (Figure S4). Whereas nicotine withdrawal did not alter the gene expression of ArgII, Asl and Ass1, theobromine significantly inhibited ArgII expression and enhanced Asl expression (Figure 8F; *p* = 0.002 and *p* = 0.0043), consistent with the RT‐PCR data (Figures 7A and 8F).

Arginine metabolism mainly occurs in the liver in mammals, and arginine can cross the blood–brain barrier to reach the CNS. For this purpose, we also detected the expression of enzymes involved in arginine metabolism in the liver and changes of arginine and NO levels in peripheral blood. In the NW group, plasma arginine levels significantly decreased compared to the control group, whereas NO levels increased. After treatment with theobromine, plasma arginine levels significantly increased, whereas NO levels decreased (Figure S5A,B). This result is consistent with findings in the cerebellum. The expression level of arginine metabolism‐related enzymes in the liver was also detected, and the mRNA level of arginase I was significantly increased in the withdrawal group, whereas the mRNA level decreased after treatment with theobromine. However, the mRNA levels of Asl and Assl did not show significant changes among the three groups (Figure S5C).

The comprehensive experimental results show that the anxiety/depression‐like behaviours caused by nicotine withdrawal are related to the activation of adenosine receptors and the arginine‐NO‐cGMP pathway. Inhibiting adenosine receptors can simultaneously inhibit the arginine‐NO‐cGMP pathway, improving emotional symptoms.

## 4 Discussion

The anxiety and depression‐like behaviours resulting from nicotine withdrawal are significant factors contributing to smoking cessation challenges and relapse [40], yet the underlying mechanisms remain unclear. Traditionally, the cerebellum is recognized for its role in coordinating and refining motor activities; however, recent evidence also implicates the cerebellum in mood regulation, particularly in anxiety and depression [21, 41]. In this study, transcriptome sequencing and untargeted metabolomics revealed that nicotine withdrawal disrupts arginine and purine metabolism in the cerebellum, correlating with anxiety and depression‐like behaviours.

Arginine, a conditionally essential amino acid, is crucial for various physiological functions, and decreased levels of arginine and its metabolites have been associated with major depressive disorder [36, 37]. In our study, levels of arginine and its related metabolites were notably decreased in the NW group, which aligns with the anxiety and depression‐like behaviours observed following nicotine withdrawal. In addition to its direct physiological effects, arginine plays a crucial role in the NO‐cGMP signalling pathway. Under the catalysis of nitric oxide synthase, arginine decomposes to produce NO, which then activates downstream signalling molecules. For example, exposure to chronic mild stress in mice results in hippocampal nNOS overexpression and depressive behaviours, which can be prevented or reversed in nNOS gene knockout mice or wild‐type mice treated with the nNOS inhibitor 7‐nitroindazole [38]. In this research, we observed elevated levels of NO and cGMP in the NW group, alongside increased expression of neuronal nitric oxide synthase (nNOS), indicating that nicotine withdrawal activates NO signalling.

In purine metabolism, significant changes included increased levels of adenosine and ITP, with decreased GDP in the NW group. The reduction in GDP levels may be associated with the increase in cGMP levels, whereas the rise in adenosine levels indicates activation of adenosine receptor signalling. Activation of A2a and A2b receptors increases cAMP production, leading to the activation of PKA downstream signalling. Conversely, activation of A1 and A3 receptors inhibits cAMP production, reduces PKA activity and enhances NO‐cGMP signalling [25]. The cAMP/PKA signalling pathway is crucial for various physiological responses, including the regulation of anxiety and depression [19, 42, 43]. Our findings indicate that nicotine withdrawal results in decreased expression of adenosine receptors A1 and A3 and increased expression of A2a. Given the elevated adenosine levels, we infer that the anxiety and depression‐like behaviours caused by nicotine withdrawal may primarily be related to the enhancement of the A2a receptor and NO‐cGMP signalling in the cerebellum.

To verify our findings, we utilized adenosine receptor inhibitors to observe the effect on nicotine withdrawal symptoms. Caffeine, a natural non‐selective antagonist for A2a and A1 adenosine receptors, has been shown to inhibit depressive behaviours [44], though it also promotes anxiety behaviours [45]. Given these complexities, we selected theobromine, a weaker adenosine receptor inhibitor compared to caffeine, for further research due to the lack of evidence linking it to anxiety disorders. Our study demonstrated that theobromine significantly alleviates the emotional symptoms associated with nicotine withdrawal. Metabolite analysis showed that theobromine treatment increased arginine levels and decreased adenosine levels, inhibiting the activation of cAMP‐PKA and NO‐cGMP signalling pathways.

Arginine metabolism primarily occurs in the liver's urea cycle. Our data showed that enzymes related to the urea cycle, such as Arg II, Asl and Ass1, are also expressed in the cerebellum. Theobromine significantly reduced the expression of Arg II while enhancing the expression of Asl. As arginine can freely cross the blood–brain barrier, we also measured arginine and NO levels in plasma, as well as the expression of arginine metabolism‐related enzymes in the liver. In the NW group, arginine levels decreased and NO levels increased, whereas theobromine treatment restored them, mirroring the cerebellar findings. Among the genes related to arginine metabolism, only Arg I expression was affected. Nicotine withdrawal reduced Arg I expression, whereas theobromine restored it. These results suggest that nicotine withdrawal impacts the entire body's arginine metabolism and NO signalling, whereas theobromine can restore these levels by inhibiting adenosine receptors. Recent studies have indicated that the urea cycle also exists in brain astrocytes, contributing to the amelioration of Alzheimer's disease [46]. Our findings suggest that theobromine may regulate the urea cycle in cerebellar astrocytes to mitigate the anxiety and depression‐like behaviours associated with nicotine withdrawal.

## 5 Conclusion

This research revealed that enhanced purine metabolism and disrupted arginine metabolism in the cerebellum contributed to the development of nicotine withdrawal affective symptoms. The non‐selective adenosine receptor antagonist, theobromine, can alleviate these symptoms by inhibiting adenosine signalling and restoring arginine metabolism in the cerebellum.

## Author Contributions

**Wenjuan Zhang:** conceptualization, methodology, data curation, writing – review and editing, visualization, investigation (equal), funding acquisition. **Yu Tian:** formal analysis, investigation, writing – review and editing. **Xiao Yang:** formal analysis, investigation, resources. **Baojiang He:** formal analysis, investigation, resources. **Haifeng Zhang:** formal analysis, investigation, resources. **Qi Zhang:** conceptualization, methodology, validation, formal analysis, investigation, writing – review and editing, supervision, funding acquisition. **Yingwu Mei:** conceptualization, validation, writing – original draft, writing – review and editing, funding acquisition.

## Conflicts of Interest

The authors declare no conflicts of interest.

## Supporting information


**Table S1.** Primers for RT‐PCR.

**Figure S1.** Supporting Information.

**Figure S2.** Supporting Information.

**Figure S3.** Supporting Information.

**Figure S4.** Supporting Information.

**Figure S5.** Supporting Information.

## Data Availability

The data that support the findings of this study are available from the corresponding author upon reasonable request.
