# Supplementary material for: Arginine Metabolism and Adenosine Receptor Signals in the Cerebellum Contribute to Nicotine Withdrawal‐Induced Anxiety/Depression‐Like Behaviours
Source: Addict Biol. 2025 Jul 30;30(8):e70076. doi: 10.1111/adb.70076 (PMC12308319; doi:10.1111/adb.70076)
Supplement: Supplementary file 1 — Table S1. Primers for RT‐PCR. [file ADB-30-e70076-s003.docx]

### Tables. Primers for RT-PCR

| Gene name | Primer-F | Primer-R |
| --- | --- | --- |
| nNOS | ACCGAGACAGGCAAATCCC | CCATCAAAGCACAGCCGAAT |
| iNOS | GGGAATCTTGGAGCGAGTTG | TGAGGGCTTGGCTGAGTGA |
| eNOS | CAGCCTAACTCCTGTCTTCCATC | GCCATCACCGTGCCCAT |
| A1 receptor | AAGCCTGGATAGCCAACGG | GGTAGATAAGAACCATGAGGAGCA |
| A2a receptor | CCCCTTCATCTACGCCTACAG | CACTCCCATTGGCCCATACT |
| A2b receptor | TCTTCCTCGCCTGCTTCG | GCTGGTGGCACTGTCTTTACTG |
| A3 receptor | GGAATAGAAAAGCAACCTTAGCG | TGTCTTGAACTCCCGTCCATAA |
| arginase II | TCCAGCCACAGGAACCCC | TGTCTGACCAAAACTTGAAGCAAT |
