# Supplementary figures and images for: Arginine Metabolism and Adenosine Receptor Signals in the Cerebellum Contribute to Nicotine Withdrawal‐Induced Anxiety/Depression‐Like Behaviours
Source: Addict Biol. 2025 Jul 30;30(8):e70076. doi: 10.1111/adb.70076 (PMC12308319; doi:10.1111/adb.70076)

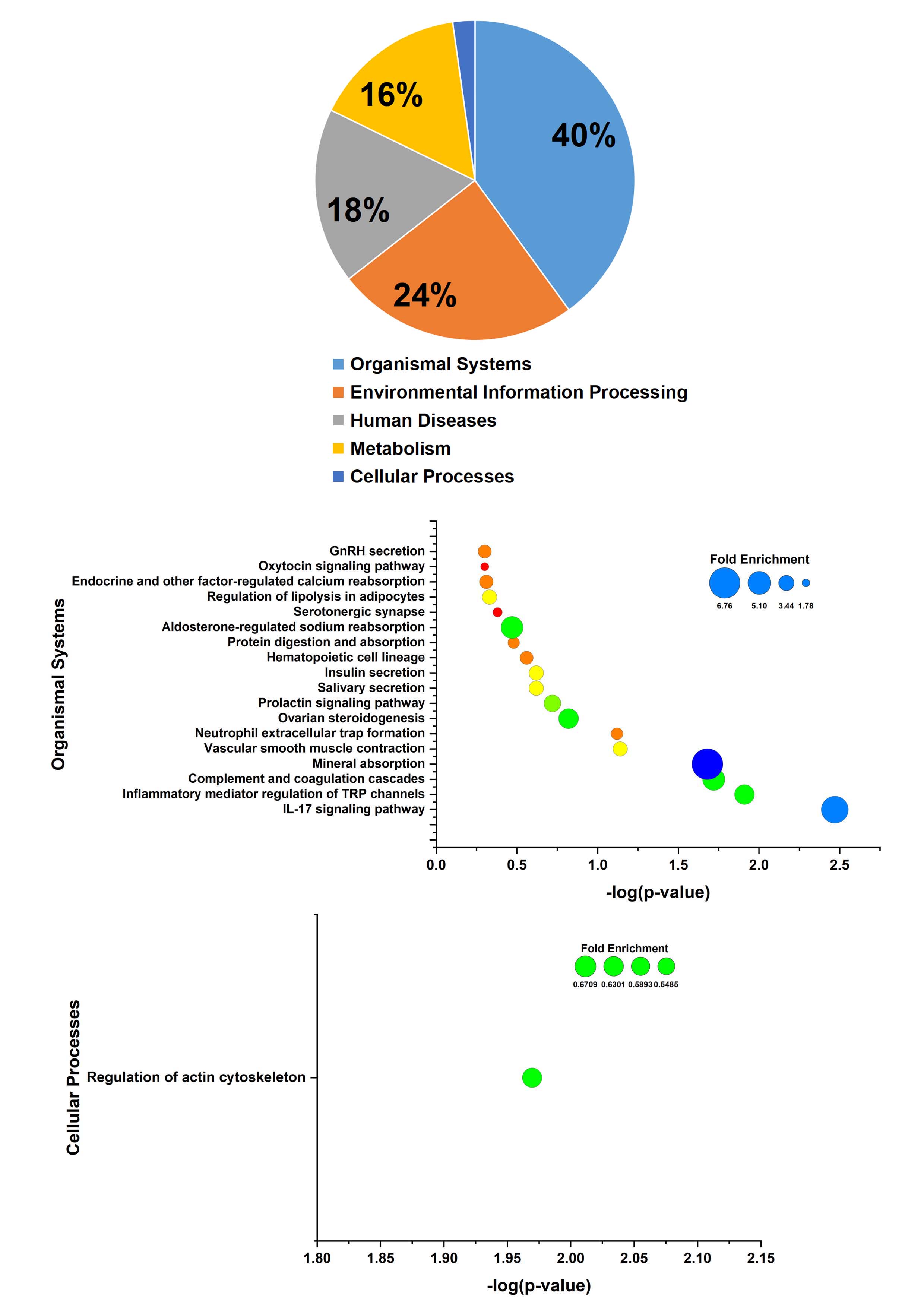

Supplement: Supplementary file 2 — Figure S1. Supporting Information. [file ADB-30-e70076-s005.jpg]

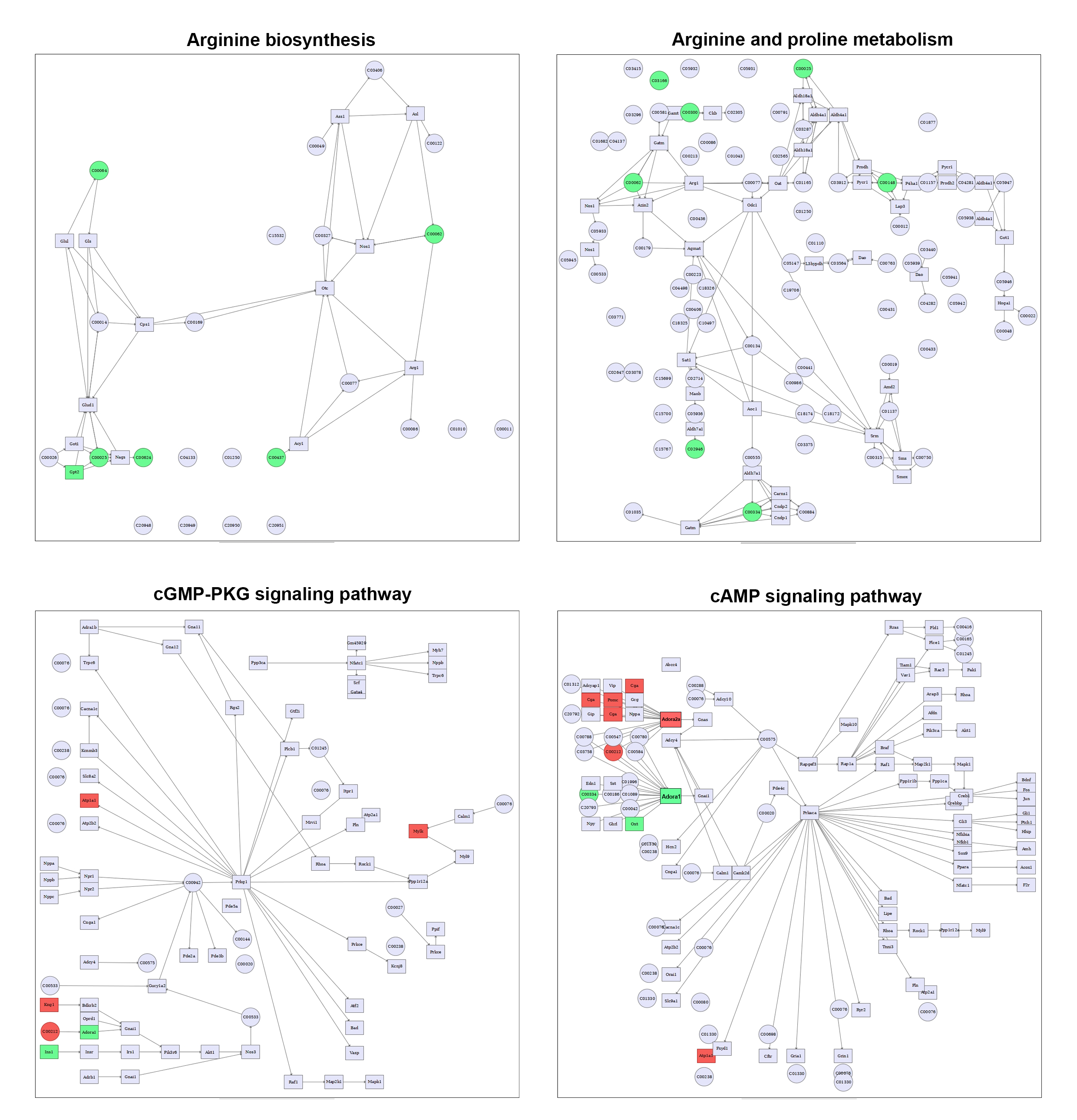

Supplement: Supplementary file 3 — Figure S2. Supporting Information. [file ADB-30-e70076-s001.tif]

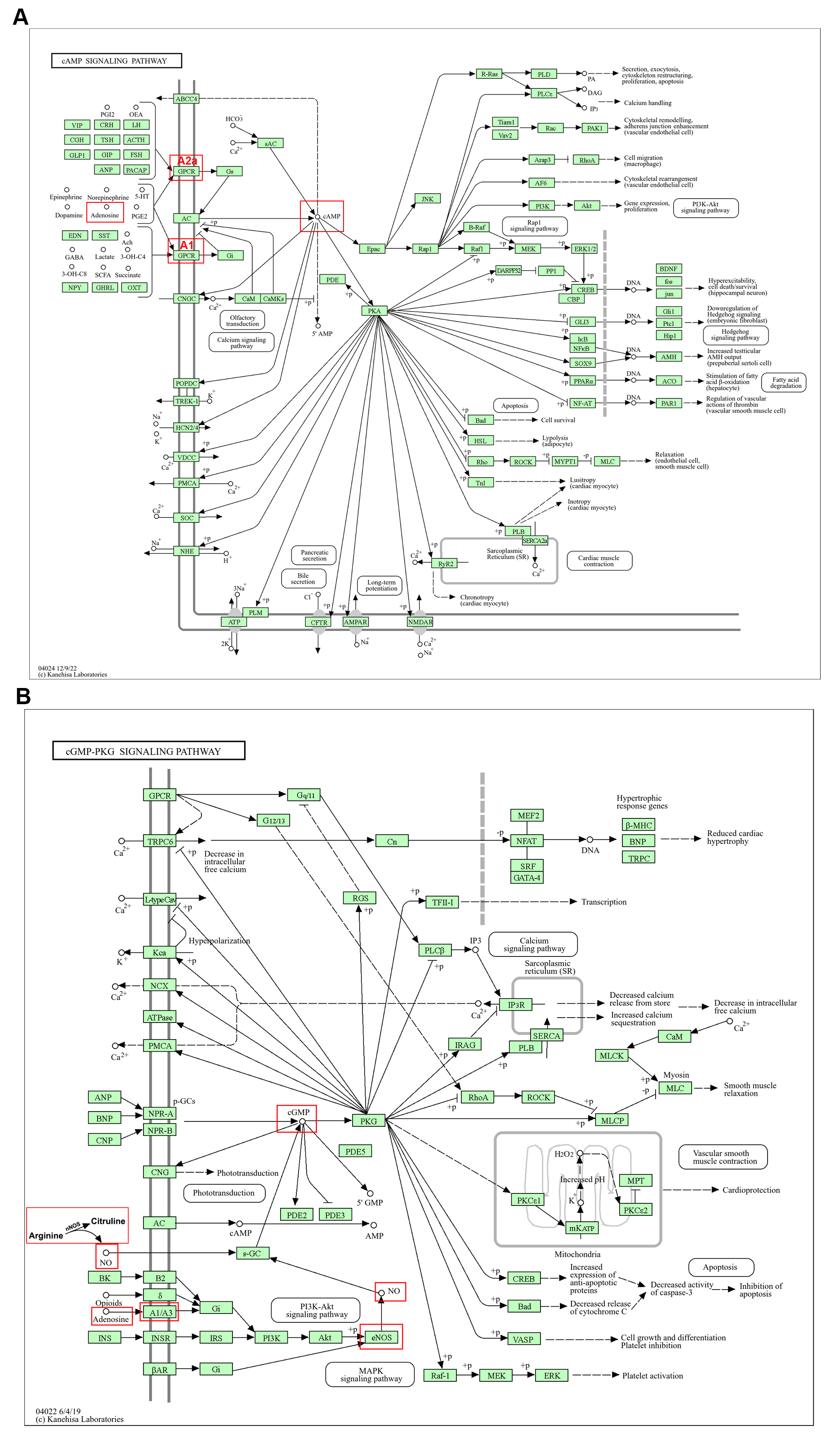

Supplement: Supplementary file 4 — Figure S3. Supporting Information. [file ADB-30-e70076-s004.tif]

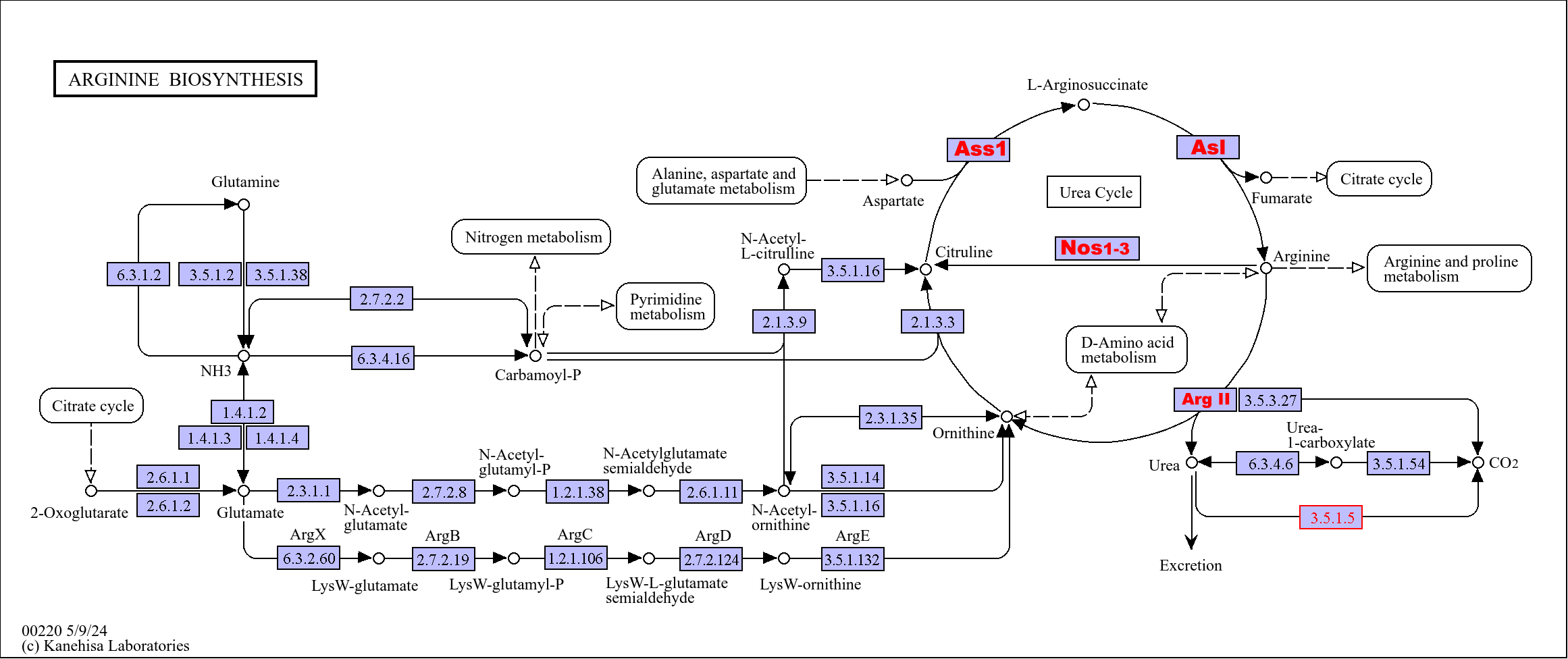

Supplement: Supplementary file 5 — Figure S4. Supporting Information. [file ADB-30-e70076-s002.tif]

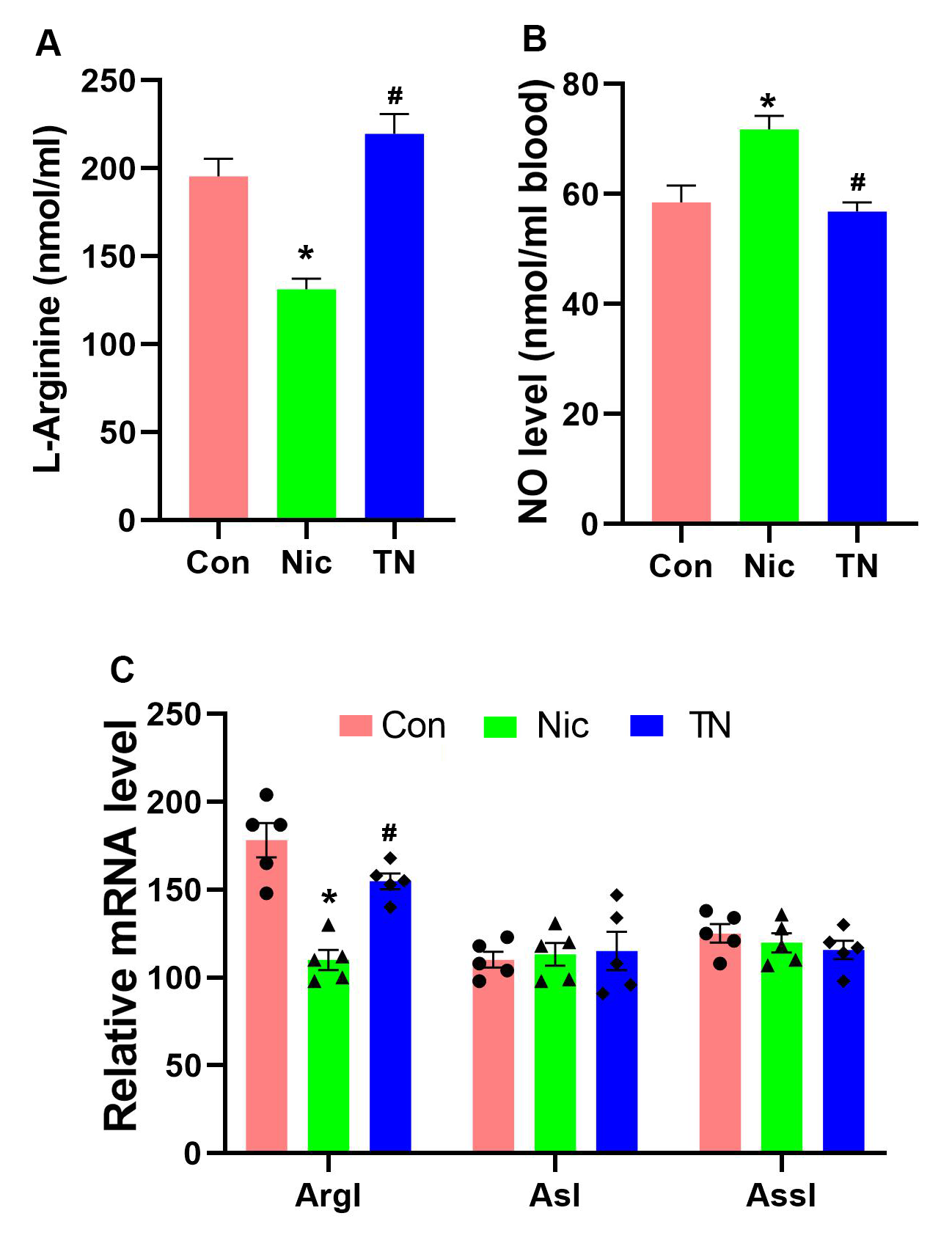

Supplement: Supplementary file 6 — Figure S5. Supporting Information. [file ADB-30-e70076-s006.tif]
